# Non-Hodgkin Lymphoma Mimicking Vestibular Schwannoma

**DOI:** 10.7759/cureus.50965

**Published:** 2023-12-22

**Authors:** Marco Antônio S Vaz, Rafaela F Gonçalves, Joel Lavinsky, Gustavo Rassier Isolan

**Affiliations:** 1 Department of Neurosurgery, The Center for Advanced Neurology and Neurosurgery (CEANNE), Porto Alegre, BRA; 2 Department of Neurology, The Center for Advanced Neurology and Neurosurgery (CEANNE), Porto Alegre, BRA; 3 Department of Morphological Sciences, Federal University of Rio Grande do Sul, Porto Alegre, BRA

**Keywords:** non-hodgkin lymphoma (nhl), vestibular schwanomas, internal auditory canal, central nervous system lymphoma, cerebellopontine angle tumor

## Abstract

Progressive unilateral hearing loss and an MRI are usually enough to diagnose vestibular schwannoma (VS). We were consulted by a 45-year-old man with otalgia and left-sided hearing loss as well as ipsilateral facial paralysis that had begun two weeks prior. Due to a possible atypical presentation of VS, an MRI was ordered, which revealed an intracanalicular lesion occupying the left cerebellopontine angle cistern. With no signs of systemic disease and considering the total left ear deafness, the patient underwent retrolabyrinthine mastoidectomy. During the procedure, a mass incompatible with VS was found and a biopsy was performed, which led to a diagnosis of non-Hodgkin’s lymphoma (NHL). The patient was referred to an oncologist for treatment and, in time, achieved complete remission of the lesion. This case shows us that symptoms of VS may vary in tumor size and location and that atypical presentations warrant investigation. Non-Hodgkin’s lymphoma (NHL), although not among the most common differential diagnoses, should be remembered due to its varied clinical presentation broadly dependent on its subtype and dissemination.

## Introduction

To diagnose vestibular schwannoma (VS), the presence of unilateral progressive hearing loss and MRI is generally sufficient. Gadolinium-enhanced MRI is the most common method for identifying and assessing the size and location of the tumor [1,2]. However, unusual cases of intracanalicular tumors, whether occupying the cerebellopontine angle cistern or not, should lead to considering broader possibilities before arriving at a differential diagnosis [3,4].

VS imaging typically reveals a well-defined lesion near or adjacent to the cerebellopontine angle. What distinguishes this from other intracranial lesions is that it is either a rounded or oval form with symmetrical features. On T1-weighted MRI, a VS appears hypointense, while on T2-weighted images, it appears hyperintense. With gadolinium contrast, VS typically demonstrates intense and homogenous enhancement due to increased blood supply to the tumor and increased permeability of tumor blood vessels [5,6].

Non-Hodgkin lymphoma (NHL) is a heterogeneous group of neoplasms originating in lymphoid tissue, mainly lymph nodes. It is characterized by the clonal expansion of malignant lymphocytes that can arise from B-cells or T-cells. The etiology of NHL is multifactorial, involving genetic, environmental, and immunological factors in its development [7,8]. Genetic alterations, such as chromosomal translocations and somatic mutations play, a significant role in the pathogenesis of specific NHL subtypes. Due to the diverse range of targets in NHL, its symptoms are extremely varied and complex. Its main symptoms are related to the systems it principally affects, but systemic symptoms include lymphadenopathy, itching, fatigue, fever, unexplained weight loss, and night sweats, among others [7-9].

This case report presents an atypical case of non-Hodgkin lymphoma that mimics VS. Differential diagnoses are discussed together with clinical aspects that raised suspicion of an atypical disease.

## Case presentation

A 45-year-old male with no previous medical history complained of occipital headache and left ear pain, along with progressive left-side facial weakness that worsened to left-side facial paralysis within one week, with no constitutional symptoms. After one more week, the patient developed ipsilateral hearing loss. The patient was initially treated with Ganciclovir by a general practitioner.

The patient, three weeks later, consulted a specialist who began empirical treatment with 20 mg of oral Prednisone every 8 hours. Under treatment, the patient experienced improvement with headaches and a slight recovery of facial paralysis within 24 hours. Continuing the use of Prednisone at the same dosage for another 48 hours resulted in the complete reversal of facial paralysis and headache, but left-sided deafness persisted, as proven by audiometry.

Cranial MRI revealed a hypointense intracanalicular lesion in the left temporal bone, which enhanced homogeneously with contrast, suggesting vestibular schwannoma (VS). When the corticosteroid was stopped, ear pain returned and the patient experienced paroxysmal episodes of facial weakness. Upon resuming corticosteroid use independently, the headache and weakness improved.

**Figure 1:**
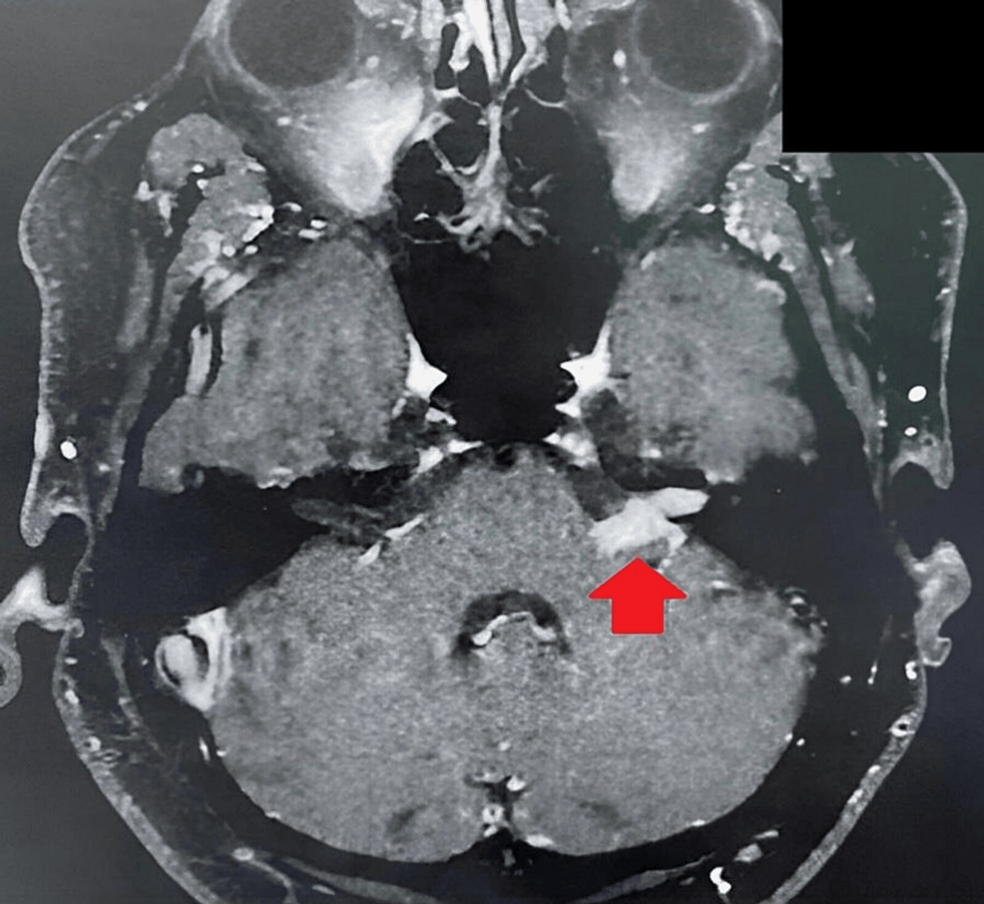
Preoperative MRI on T1 showing an expansive lesion with enhancement after gadolinium administration located along the pathway of the left internal auditory canal and cerebellopontine angle, measuring approximately 1.4 cm on the laterolateral axis. The red arrow shows the tumor.

Due to the atypical presentation of VS, another MRI was performed 15 days after the initial examination, a time at which the patient wasn't using steroids. The MRI showed that the previously intracanalicular lesion was now occupying the cerebellopontine angle cistern on the left side (Figure 1). With sarcoidosis being a possibility, the serum level of angiotensin-converting enzyme and a chest tomography were taken, but both came back normal. Routine laboratory and cerebrospinal fluid tests also showed normal results.

**Figure 2:**
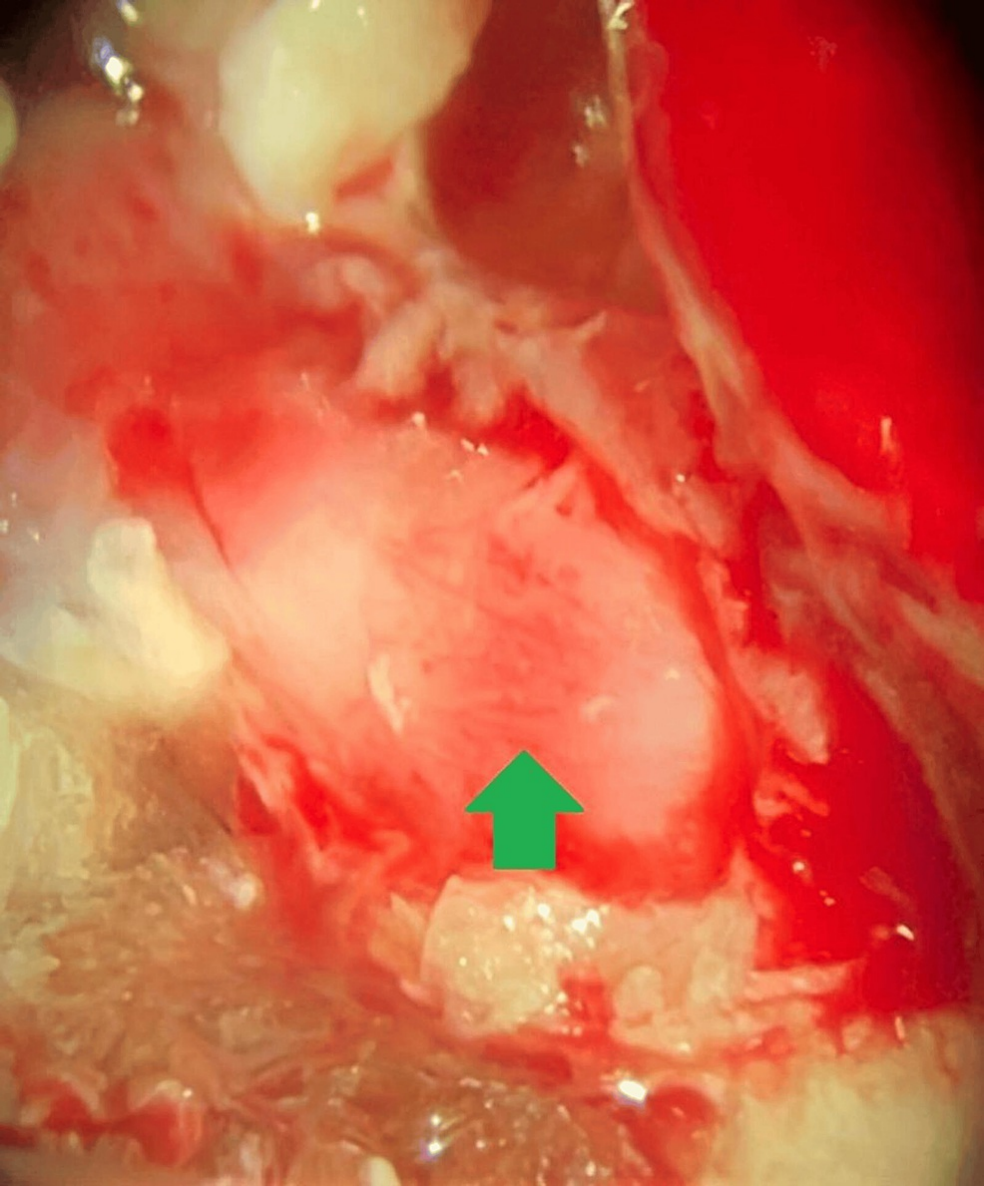
Intraoperative view via the retrolabyrinthine approach showing the lesion occupying the left internal auditory canal. The green arrow shows the tumor.

In the absence of any signs of systemic disease and considering the total deafness in the left ear based on audiometry, the patient underwent mastoidectomy via the retrolabyrinthine approach (Figure 2). This approach allowed easier differentiation of vestibular and facial schwannoma and offered a better view of the lesion. During the procedure, a whitish mass with a smooth surface was found after assessing the internal auditory canal. The macroscopic aspect of the lesion was different from those usually associated with VS. Furthermore, facial nerve motor evoked potentials decreased at the slightest manipulation of the tumor. Thus, VS was ruled out and, based on the intraoperative findings, the diagnosis of facial nerve schwannoma was proposed. For this reason, an incisional biopsy was performed instead of whole tumor extraction. Immediate postoperative assessment of facial function showed facial nerve preservation.

**Figure 3:**
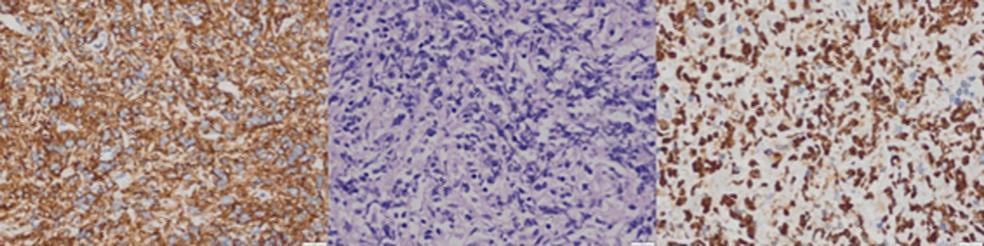
The figure shows that the tumor was highly positive for Ki67 (left image) and CD 20 (right image) with the hematoxylin and eosin (H&E) method (central image)

The pathological and immunohistochemical findings were as follows: high-grade non-Hodgkin lymphoma and a possibly diffuse large B-cell lymphoma as shown in histopathological slide photos (Figure 3).

Post-diagnosis, a positron emission tomography (PET) scan was performed, which did not show any more lesions in other parts of the body. The patient was referred to an oncologist for treatment with methotrexate and vincristine followed by an autologous bone marrow transplant, which led to complete remission, associated with a decrease in kidney function and no neurological deficits.

## Discussion

VS is the most common tumor affecting the internal auditory canal and cerebellopontine angle, amounting to 85% of diagnoses. It is a benign, slow-growing tumor derived from Schwann cells that surround the vestibular nerve and is mostly unilateral [1,5].

Symptoms associated with VS can vary considerably depending on the size and location of the tumor. Initial symptoms may include progressive hearing loss in one ear, tinnitus, ear fullness, and balance disorders such as dizziness and vertigo [1-3]. As the tumor grows, it may compress adjacent structures, such as the facial nerve, and lead to facial weakness or paralysis [6].

A diagnosis of VS is made by evaluating medical history, including physical examination, audiological tests, and imaging studies such as MRI. Audiometry shows asymmetric neurosensory hearing loss and greater impairment of speech discrimination than would be expected for the degree of hearing loss [5,6]. Treatment is determined by various factors, including tumor size, location, patient's age, symptoms present, and more [1-6].

Considering the variable symptoms of VS, it is essential to consider differential diagnoses. Some of them are:

Meningioma

These are benign tumors that originate from the meninges. They can occur in the cerebellopontine angle, similar to VS, and may present similar symptoms such as hearing loss, tinnitus, and vertigo. Sometimes, differentiation from VS is made with features found in imaging such as the dural tail sign [10].

Brain Metastases

Metastases from primary tumors in other parts of the body can spread to the brain and cause symptoms similar to those of VS. Previous cancer history, presence of lesions in other organs, and cerebrospinal fluid test can help differentiate brain metastases from a schwannoma [11,12].

Facial Nerve Schwannoma

This tumor arises from the facial nerve, which controls facial movements. Depending on the location and size of the tumor, it can present symptoms similar to VS such as facial weakness and difficulty closing the eyes [13].

Sarcoidosis

A multisystemic granulomatous disorder characterized by the formation of non-caseating granulomas. The disease primarily affects the lungs and intrathoracic lymph nodes, but it can also involve the central nervous system. Sarcoidosis manifests with heterogeneous clinical presentations, ranging from asymptomatic cases, identified incidentally, to progressive forms marked by respiratory insufficiency and organ dysfunction. In cases that attack the CNS, hearing loss, tinnitus, and vertigo can also be symptoms [14].

In addition to the previously mentioned differential diagnoses, non-Hodgkin lymphoma (NHL), should also be considered even though it is rare. NHL is a malignant hematological neoplasm originating from B-cells, T-cells, or natural killer (NK) cells [7,8]. Unlike Hodgkin's lymphoma, NHL does not exhibit the characteristic Reed-Sternberg cells. Instead, it represents a diverse group of diseases with varied genetic and molecular characteristics. The etiology of NHL is still uncertain, but multifactorial aspects, such as immunodeficiency, viral infections, exposure to toxic chemicals, and genetic predisposition, may play a role in its pathogenesis [7].

The pathophysiology of NHL is not fully understood, but it is believed that genetic alterations in lymphocytes contribute to its development, stimulating uncontrolled proliferation and preventing apoptosis. This results in the formation of tumors in lymph nodes or multifocal lymphoid tissues [8,9]. In the CNS, the exact pathogenesis of their neurotropism is debatable, however, the transformation during hematogenous dissemination can lead to new surface markers to “home” inside the CNS [15].

The clinical presentation of NHL varies widely depending on the subtype and spread of the disease. Common symptoms include painless lymphadenopathy (enlarged lymph nodes), weight loss, fever, night sweats, fatigue, and abdominal pain in cases of gastrointestinal involvement. Additionally, due to potential dissemination, unilateral deafness and headache can also be found [7-9,16].

The diagnosis of NHL requires an integrated approach combining clinical findings, advanced imaging studies (e.g., computed tomography, MRI, PET scan), and histopathological analyses [9].

Treatment for NHL is highly individualized, considering the specific subtype, stage, age, and clinical condition of the patient. Therapeutic options include chemotherapy, radiation therapy, immunotherapy, targeted therapy, and hematopoietic stem cell transplantation. Emerging immunotherapies, such as monoclonal antibodies and chimeric antigen receptor T-cell therapy (CAR-T), have shown promise in selected subtypes [9,17].

The prognosis of NHL varies widely among different subtypes and stages of the disease. Some cases are indolent and respond favorably to treatment while others can be aggressive and present significant therapeutic challenges [8,16].

## Conclusions

This case report illuminates the diagnostic challenges associated with intracranial lesions, specifically differentiating between vestibular schwannoma (VS) and an atypical presentation of high-grade non-Hodgkin lymphoma (NHL). The intricate interplay of clinical symptoms, imaging findings, and histopathological analyses underscores the complexity inherent in neuro-oncological diagnoses.

Beyond the conventional paradigms of VS, the case accentuates the imperative for clinicians to exercise clinical acumen, considering a spectrum of rare differentials when faced with evolving clinical presentations. And illuminate the imperative for more extensive investigations when confronted with atypical presentations of vestibular schwannoma (VS).
